# Supplementary figures and images for: From 3D to 3D: isolation of mesenchymal stem/stromal cells into a three-dimensional human platelet lysate matrix
Source: Stem Cell Res Ther. 2019 Aug 9;10:248. doi: 10.1186/s13287-019-1346-2 (PMC6688329; doi:10.1186/s13287-019-1346-2)

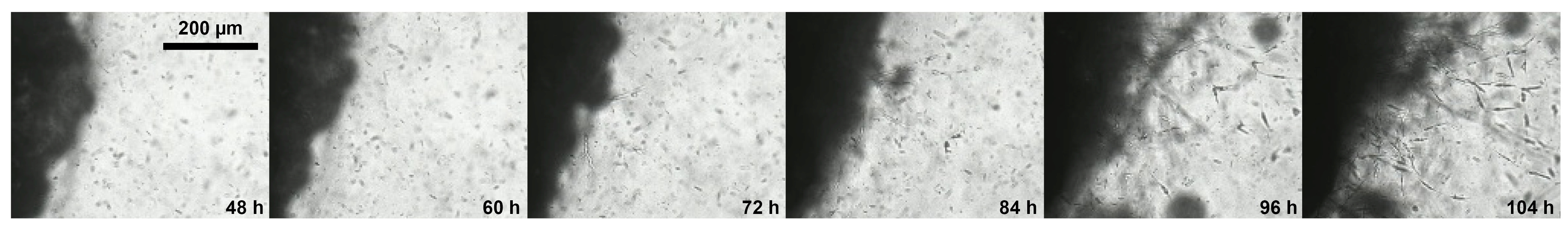

Supplement: Supplementary file 1 — Figure S1. Time series of micrographs of adipose tissue embedded in PLMatrix over a course of 104 h. Cells migrating from the tissue into the matrix can be observed after 72 h. (JPG 1352 kb) [file 13287_2019_1346_MOESM1_ESM.jpg]

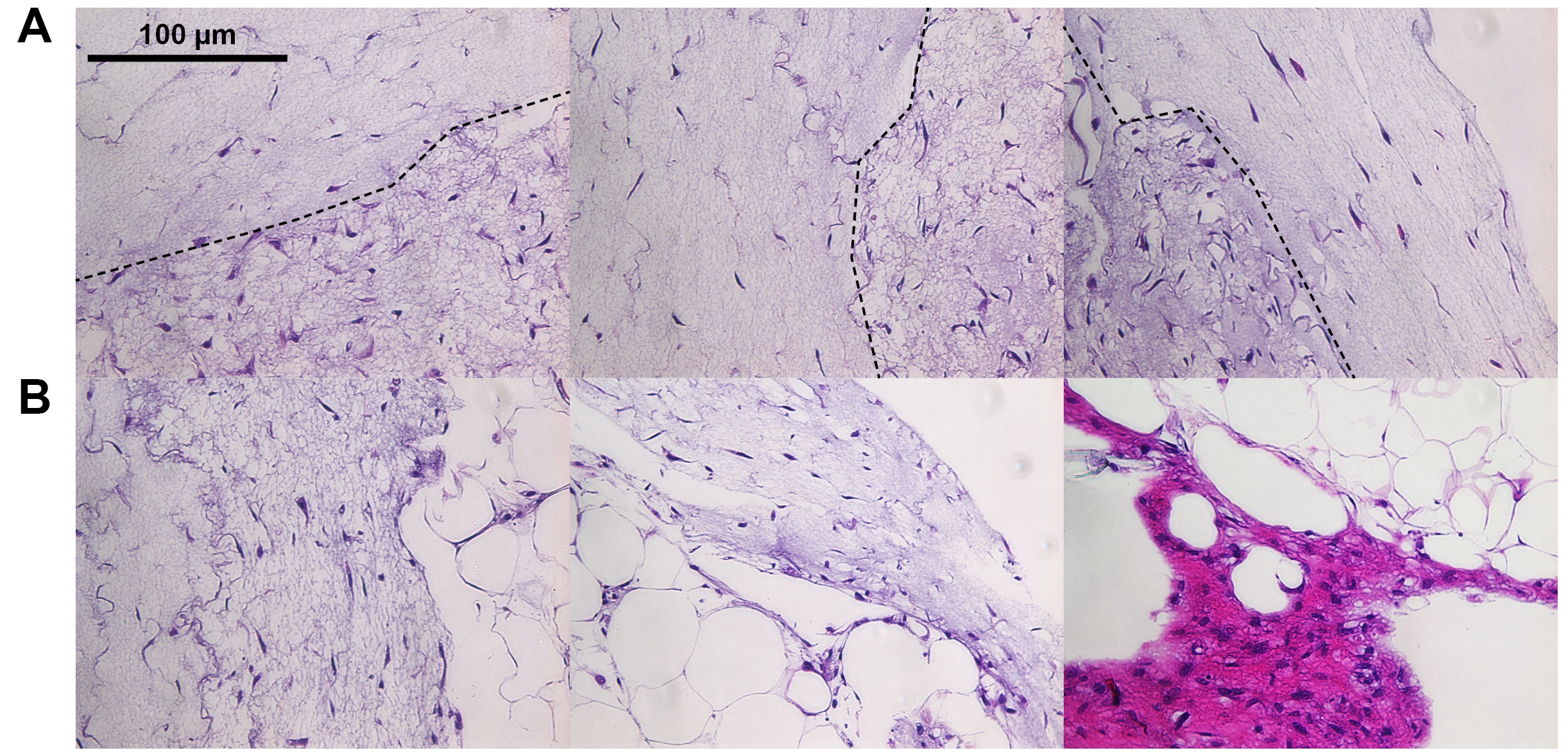

Supplement: Supplementary file 3 — Figure S2. Histological sections stained with hematoxylin (blue, stains for nuclei) and eosin (pink, stains for collagen). (A) PLMatrix with MSCs that migrated into the hydrogel (the dashed line separates areas where MSCs started to remodel the matrix and areas that are infiltrated by only a few cells). (B) Micrographs covering actual areas of MSC outgrowth from adipose tissue into PLMatrix. (JPG 2257 kb) [file 13287_2019_1346_MOESM3_ESM.jpg]

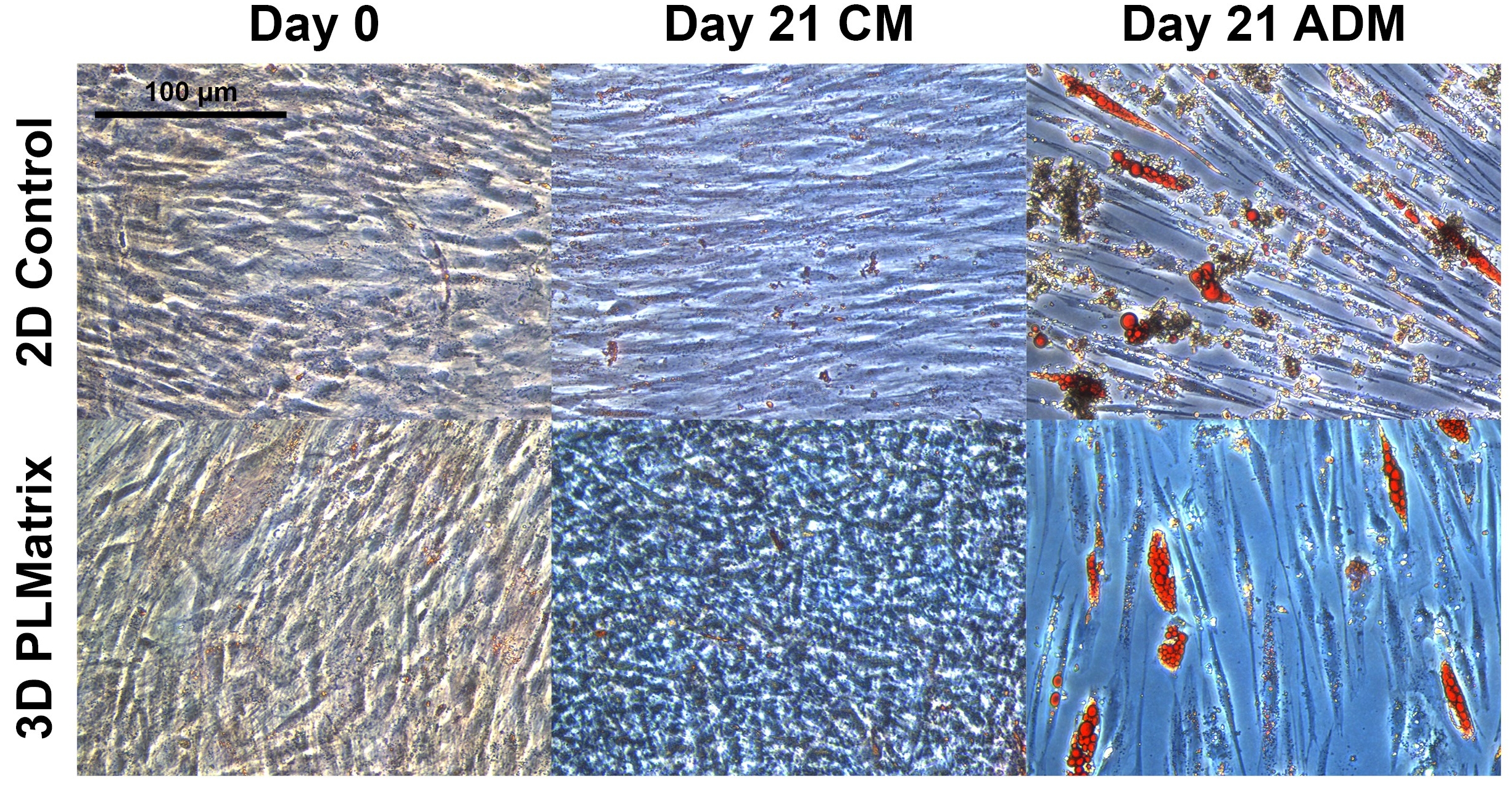

Supplement: Supplementary file 4 — Figure S3. Cells derived from isolation by enzymatic treatment (2D control) or from 3D isolation (3D PLMatrix) cultivated for 0 or 21 days in control medium (CM) or adipogenic differentiation medium (ADM) stained with Oil Red O. (JPG 2802 kb) [file 13287_2019_1346_MOESM4_ESM.jpg]

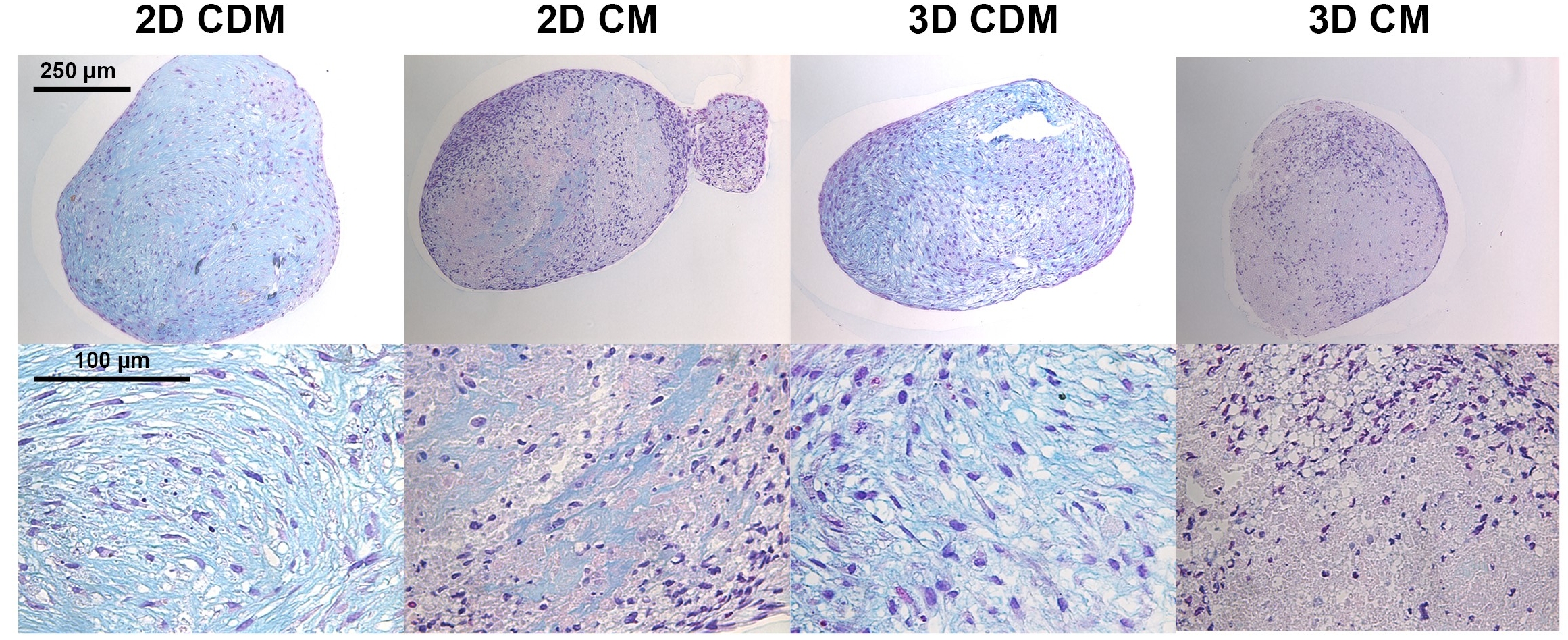

Supplement: Supplementary file 5 — Figure S4. Histological sections of cells derived from isolation by enzymatic treatment (2D control) or from 3D isolation (3D PLMatrix) cultivated for 21 days in control medium (CM) or chondrogenic differentiation medium (CDM) stained with Alcian Blue. (JPG 1693 kb) [file 13287_2019_1346_MOESM5_ESM.jpg]

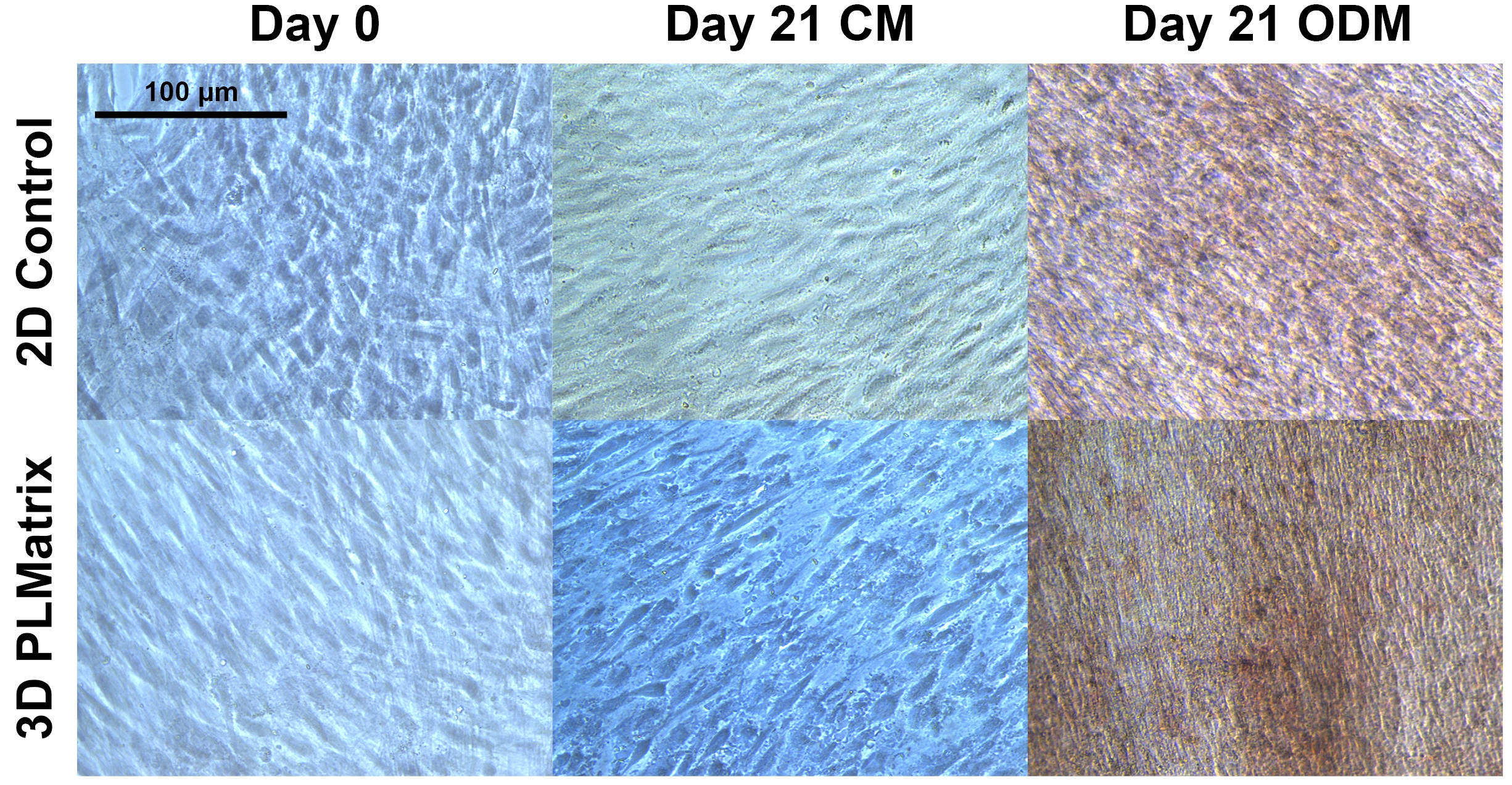

Supplement: Supplementary file 6 — Figure S5. Cells derived from isolation by enzymatic treatment (2D control) or from 3D isolation (3D PLMatrix) cultivated for 0 or 21 days in control medium (CM) or osteogenic differentiation medium (ODM) stained with Alizarin Red. (JPG 2645 kb) [file 13287_2019_1346_MOESM6_ESM.jpg]
